# Genetic counseling as preventive intervention: toward individual specification of transgenerational autism risk

**DOI:** 10.1186/s11689-021-09389-8

**Published:** 2021-09-16

**Authors:** Natasha Marrus, Tychele N. Turner, Elizabeth Forsen, Drew Bolster, Alison Marvin, Andrew Whitehouse, Laura Klinger, Christina A. Gurnett, J. N. Constantino

**Affiliations:** 1 Department of Psychiatry, Washington University School of Medicine, 660 S. Euclid Ave; Box 8504, St. Louis, MO 63110 USA; 2 Department of Genetics, Washington University School of Medicine, 660 S. Euclid Ave; Box 8108, St. Louis, MO 63110 USA; 3 Maryland Center for Developmental Disabilities, Kennedy Krieger Institute, PACT Building/Office 121B; 7000 Tudsbury Road, Baltimore, MD 21244 USA; 4 Telethon Kids Institute, Perth Children’s Hospital, 15 Hospital Avenue, Nedlands, WA 6009 USA; 5 TEACCH Autism Program, Department of Psychiatry, University of North Carolina at Chapel Hill, Campus Box #7180, Chapel Hill, NC 27599-7180 USA; 6 Department of Neurology, Washington University School of Medicine, 660 S. Euclid Ave; Box 8111, St. Louis, MO 63110 USA

**Keywords:** Personalized medicine, Genetic counseling, Family studies, Reproductive health planning, Early detection

## Abstract

**Background:**

Although autism spectrum disorders (ASD) are among the most heritable of all neuropsychiatric syndromes, most affected children are born to unaffected parents. Recently, we reported an *average* increase of 3–5% over general population risk of ASD among offspring of adults who have first-degree relatives with ASD in a large epidemiologic family sample. A next essential step is to investigate whether there are measurable characteristics of individual parents placing them at higher or lower recurrence risk, as this information could allow more personalized genetic counseling.

**Methods:**

We assembled what is to our knowledge the largest collection of data on the ability of four measurable characteristics of unaffected prospective parents to specify risk for autism among their offspring: (1) sub clinical autistic trait burden, (2) parental history of a sibling with ASD, (3) transmitted autosomal molecular genetic abnormalities, and (4) parental age. Leveraging phenotypic and genetic data in curated family cohorts, we evaluate the respective associations between these factors and child outcome when autism is present in the family in the parental generation.

**Results:**

All four characteristics were associated with elevation in offspring risk; however, the magnitude of their predictive power—with the exception of isolated rare inherited pathogenic variants —does *not* yet reach a threshold that would typically be considered actionable for reproductive decision-making.

**Conclusions:**

Individual specification of risk to offspring of adults in ASD-affected families is not straightforwardly improved by ascertainment of parental phenotype, and it is not yet clear whether genomic screening of prospective parents in families affected by idiopathic ASD is warranted as a clinical standard. Systematic screening of affected family members for heritable pathogenic variants, including rare sex-linked mutations, will identify a subset of families with substantially elevated transmission risk. Polygenic risk scores are only weakly predictive at this time but steadily improving and ultimately may enable more robust prediction either singly or when combined with the risk variables examined in this study.

## Background

Among neurodevelopmental disorders, autism spectrum disorder (ASD) is distinguished by a high degree of heritability, on the order of 85% [1]. Although many *sporadic* cases of ASD have now been associated with rare, highly-penetrant de novo pathogenic variants [2, 3], *inheritance* of the condition is believed to be mediated principally by polygenic risk, i.e., the aggregation of numerous common allelic variants, each individually of negligible main effect [4, 5]. In support of this model, rare, deleterious de novo variants are more frequently observed in simplex families with a single sporadic case of ASD versus multiplex families with multiple cases of ASD [6], and family members in multiplex families exhibit higher levels of sub clinical quantitative autistic traits (QATs) relative to simplex families [7, 8], consistent with higher polygenic liability. In a minority of cases (the exact proportion as yet unknown), ASD transmission in families has also been linked to the segregation of a rare inherited genetic variant of major effect [9], which may be of maternal or paternal origin, and emerging findings suggest both that (1) genetic liability can reflect contributions of common and rare variant risk [6] and (2) that rare variants and polygenic risk factors exert additive effects [10].

Many genes affected by ASD-associated mutations, which are also implicated in other neurodevelopmental disorders [11, 12], converge in functionally interrelated pathways [13, 14] regulating neuronal development, synaptic signaling, chromatin remodeling, and transcription [15], and recently, both rare inherited and rare de novo risk factors were found to contribute to a common protein-protein interaction network [16]. A conundrum in the prediction of ASD recurrence in families is that despite the strong heritability of the condition, most children with autism are born to unaffected parents. The increasingly recognized intersections between contributions of rare and common variants to ASD’s inherited liability and underlying neurobiology suggests that identifying measurable parental characteristics linked to these factors (e.g., ASD trait liability, genetic profiles) could provide more individualized specification of transgenerational ASD risk, with clinical implications for genetic counseling.

Recently, we attempted to quantify the elevation in ASD risk (i.e., over the general population average) in “second-generation” offspring to parents with and without close relatives with ASD. This is of particular public health relevance because a generation of siblings of the individuals who comprised the wave of increased prevalence of ASD, which now stands at 1 in 54 children in the U.S. (1.9%) [17], have reached child-bearing age. Based on a large, epidemiologically-ascertained two-generation family sample in Sweden (*n* > 800,000), we observed that the risk of ASD among children whose parents had an ASD-affected sibling was two times higher than the population average for fathers and three times higher for mothers [18]. Given that these relative risk elevations represent a population average, a next essential step is to investigate whether there are measurable characteristics of individual parents placing them at higher or lower transgenerational risk to their offspring. That is, while the overall offspring risk for a mother with an ASD-affected sibling is 5.5% [18], determining how to specify a particular parent’s increased risk within the overall population distribution would lead to actionable options for reproductive decision-making via pre-conceptional genetic counseling.

Here we assemble data on four established correlates of familial recurrence of autism for their ability to make individual-specific predictions about accentuation or attenuation of ASD risk to the offspring of adults in families affected by autism. The four correlates are (1) sub clinical autistic trait burden, (2) parental history of a sibling with ASD, (3) transmitted molecular genetic variants, and (4) parental age. Sub clinical autistic traits, which are correlated between parents and offspring [19, 20], are known to aggregate in the unaffected siblings of individuals with autism [7, 21] and when elevated in both parents to raise the average risk of autism to offspring [19]. Most molecular genetic variants unequivocally associated with autism are de novo, and therefore not transmitted from healthy parents to affected children. However, inherited variants of lower pathogenicity may contribute to autism and have been implicated in silent transmission through an unaffected parent [22]. Finally, advancing paternal age, implicated as a risk factor for neurodevelopmental disorders, including ASD [23–26], has been associated with accumulation of de novo variants in male sperm [27, 28], which may amplify ASD risk to offspring.

Our appraisal of these four candidates for individual specification of pre-conceptional risk to offspring for transmission of familial autism harnessed a diverse set of existing and newly collected research datasets. These datasets, which included prospective and quasi-prospective study designs, ascertained one or more of these candidate predictors in the setting of contrasting intergenerational transmission patterns. Briefly, the datasets comprised parents (primarily mothers) in families clinically ascertained for autism in either the parental or the offspring generation. Quantitative Autistic Traits (QATs) in mothers were compared across scenarios that inferred higher or lower likelihood of inherited ASD liability being transmitted to offspring *through the mother*. These contrasting scenarios entailed the following: (a) mothers of single versus multiple ASD-affected offspring, (b) mothers within pedigrees consistent or not consistent with silent maternal transmission of ASD risk, (c) mothers of ASD-affected offspring with versus without an ASD-associated de novo (non-inherited) genetic variant, and (d) mothers with an ASD-affected sibling and offspring with versus without ASD. In a separate clinical sample, we compared the burden of inherited chromosomal rearrangements, ascertained by chromosomal microarray, in mothers and offspring from pedigrees with or without a maternal family history of ASD. Finally, among parents with one ASD-affected child (i.e., from simplex families), we examined whether there existed a relationship between parental age and the burden of ASD*-*associated de novo variants.

## Methods

Data were drawn from (1) a quasi-prospective study of mothers with siblings affected by ASD, (2) a genomically characterized sample of clinic patients and research participants, and (3) several large datasets described in previous publications that contained subsets of families informative with respect to the candidate parental predictors of transgenerational ASD risk described above (summarized in Table 1). Informativeness was determined by (1) whether mothers had one or more systematically ascertained candidate predictors (i.e., quantitative autistic traits (QATs), personal history of an ASD-affected sibling, microarray genotype, and maternal age) and (2) whether the family of a mother within the dataset represented one of the two intergenerational transmission patterns that were specifically being compared in a given analysis.

**Table 1:** Descriptions of analyses and study samples

Analysis	Study samples						
	New analysis of existing data				New data		
	Simons Simplex Collection		Autism Genetic Resource Exchange	WUSTL Family Studies	2nd Generation Project		WUSTL Genomic Sample
	Mothers	Fathers			Unaffected mothers	Offspring	
1a. Maternal QATs* in simplex versus multiplex families	*N*=2839		*N*=52	*N*=245			
1b. Maternal QATs* in pedigrees consistent or not consistent with silent maternal transmission				*N*=88	*N*=8		
2. Parental QATs* in simplex families with/without de novo variants in ASD-affected offspring	*N*=2851	*N*=2851					
3. Maternal QATs* for unaffected sisters of ASD-affected sibling with/without ASD-affected offspring					*N*=41		
**4. Offspring ASD diagnosis for unaffected mothers with ASD-affected sibling**					*N*=113	*N*=220	
5. Maternally inherited variants in ASD-affected offspring with/without maternal family history of ASD							*N*=103
**6a. Parental age in simplex families relative to number of de novo variants in ASD-affected offspring**	*N*=2140	*N*=2140					
**6b. Parental age in simplex families relative to number of de novo variants in unaffected siblings of ASD-affected offspring**	*N*=1605	*N*=1605					

Study samples are listed according to whether they were derived from existing or new data collections. Rows list analyses based on their order in the text. Numbers of participants from each sample for a given analysis are listed by column headings. Bolded analyses were statistically significant (*p*<.05)

*WUSTL* Washington University in St. Louis, *QATs* Quantitative Autistic Traits

*All QATs were measured with the Social Responsiveness Scale-2 [29]

### New data collection

*Second Generation Project:* This family study employed a “quasi-prospective design” in which parents of a child with ASD provided both QAT phenotyping on their unaffected adult-aged daughters, who were sisters of an individual with ASD, and history regarding ASD diagnoses in these sisters’ offspring, who were their grandchildren in the second generation. The study design was quasi-prospective in that ascertainment of index cases of ASD in the parental generation was the basis for data collection on diagnostic outcomes in second-generation grandchildren. As the recruited informants, grandparents offered the advantage of long-term knowledge of their daughters’ (i.e., the unaffected sisters’) QATs, which would be expected to show temporal stability [30], while avoiding selection bias that could have occurred with recruitment of unaffected sisters. To establish cases of ASD in their grandchildren, grandparents were asked to report only diagnoses made by a physician, psychologist, or school-based evaluation. Grandparents were also asked whether they were aware of any suspicion for an ASD diagnosis. The study was motivated by public health concerns related to ASD cross-generational transmission in unaffected sisters of an individual with ASD, who according to a female protective effect hypothesis [31], would be likely to silently transmit amplified risk of ASD to their offspring. A multisite recruitment involved four sites: the Interactive Autism Network (IAN), the Telethon Kids Institute (TKI) in Perth, Western Australia, the TEACCH Autism Program at the University of North Carolina, and a local community-based recruitment from Washington University in St Louis (WUSTL). IAN was selected due to its large size (over 25,000 families) and relatively unbiased online ascertainment. Both TKI and TEACCH had established populations of families with adult-aged children with ASD: TKI has served one of the oldest autism family cohorts on the continent of Australia, in a city with low levels of migration, while TEACCH, at the University of North Carolina, included a cohort of 279 families with an adult-aged family member with ASD. This family member was previously diagnosed by TEACCH during childhood and families had been re-contacted in middle adulthood to characterize long-term trajectories. Recruitment was initiated by email (IAN), mail (TEACCH, WUSTL), or telephone (TKI, TEACCH, WUSTL) contact, with a focus on families whose index child with ASD and/or known siblings of the proband were adult-aged. Recruitment included fliers, letters, or emails to potentially eligible families identified from clinical or research participant databases, as well as word-of-mouth and television interviews regarding the study. The enrollment process included confirmation that the families were not co-enrolled at more than one site. Following enrollment and consent, grandparents first completed a brief online questionnaire regarding their family history of ASD. Grandparents were then re-contacted regarding the opportunity to consent to an extended study protocol involving a telephone interview to confirm family history and pedigree structure and completion of the Social Responsiveness Scale-2 (SRS-2; see below) on mothers of second-generation offspring (i.e., their unaffected daughters who had a sibling with ASD). The proportion of second-generation offspring with ASD did not differ for families who participated solely in the brief versus extended protocol (*x*^2^(1) = 0.05, *p* =.83). Study procedures were approved by the Washington University in Saint Louis Institutional Review Board (#201508067), as well as the Institutional Review Boards for the John Hopkins Medicine IRB (IAN), TKI, and University of North Carolina (TEACCH).

*Washington University Genomic Dataset*: We assembled results of microarray-based genomic characterization (see below) acquired among ASD-affected children and their mothers through routine clinical care at the WUSTL Autism Clinical Center or by virtue of co-enrollment in HD 087011, which recruited women from the community enriched for asymptomatic transmission of ASD risk [32]. For mothers of ASD-affected children from the clinical service, microarray testing was performed if a chromosomal variant was first identified in the proband, given the testing was conducted as part of the child’s medical work-up. The combined sample comprised (i) 73 mothers who did not have a sibling or parent with autism, but had one child with autism (i.e., a simplex autism case) and (ii) 30 mothers of children with a familial recurrence of autism in which the pedigree structure was suggestive of maternal inheritance (e.g., mothers with an ASD-affected sibling or mothers of concordant ASD-affected half-siblings). All were informative with respect to the question of whether autism was associated with a maternally inherited chromosomal rearrangement on the basis of one of the following: (a) the affected child was tested by microarray and found to be *negative for a pathogenic chromosomal rearrangement*, (b) the affected child was tested by microarray and found to be *positive* for a pathogenic chromosomal rearrangement AND the mother was tested to determine whether the rearrangement was inherited, and (c) a mother of a child with ASD in group ii above was tested by chromosomal microarray.

### Existing data sources for secondary analysis

*Autism Genetics Network (MH100027)*: This dataset (2009–2018) involved exclusively African American children with ASD and their families recruited from clinical services and community agencies serving individuals with ASD and are described in detail in [33]. Among families recruited at Washington University in St. Louis, Missouri (*n*=205), maternal SRS-2 scores were available in 149 families.

*Longitudinal Study of Quantitative Autistic Traits* (HD042541): This study (2003–2014) characterized the longitudinal course of autistic traits in ASD-affected children who had at least one full biological sibling and who were consecutively ascertained through a child psychiatry clinic. Complete details of the dataset are provided in [30]; maternal SRS-2 scores were available in 84 families.

*Early Reciprocal Social Behavior Study*: This study [34] investigated the early development of reciprocal social behavior, an aspect of social competency disrupted in ASD. Data were included from this study’s clinical sample of children ages 18 to 36 months with a suspected diagnosis of ASD or community diagnosis of ASD by a physician. These participants were recruited from local clinics and community postings. Maternal SRS-2 scores were available in 12 families.

*Autism Registry Collections*: Data were obtained from the Autism Genetic Resource Exchange (AGRE [35];) as well as the Simons Simplex Collection (SSC [36];). These repositories contain genetic data, biomaterials, and behavioral phenotyping from families who have children affected by ASD. From AGRE, we included maternal SRS-2 data available within a group of *multiplex* families (*n*=210) who were initially recruited to provide QAT phenotyping on their offspring [7]. Maternal SRS-2 ratings (*n*=52) were collected in a follow-up data collection within this sample. Among the participants whom we were able to trace and re-contact, we observed a high response rate, and there were no statistically significant differences in the means and distributions of SRS-2 scores of probands or their male siblings between families who did versus did not provide maternal QAT assessments. From the SSC, which includes only simplex families with one ASD-affected child, we utilized data from families with maternal and paternal QAT ratings and genotypic characterization of ASD probands (*n*=2839). Pathogenic de novo variants for ASD were defined as the following: (a) protein-coding de novo CNVs, as described in Sanders et al., 2015, where variants were identified by microarray [37] and (b) protein-coding de novo missense and loss-of-function mutations from Wilfert et al., 2021 [38], where variants were identified based on whole genome sequencing, with restriction to the 253 significant genes for neurodevelopmental disorders from Coe et al. [39].

### Measures

*Social Responsiveness Scale-2 (SRS-2)*: Parental, spousal, or self-report data on quantitative autistic traits was acquired by ratings using the SRS-2, a quantitative measure of autism-related variation in reciprocal social behavior [40]. Use of the adult version of the scale has been described extensively in prior reports [19, 20] and in the SRS-2 manual [41]. The instrument’s internal consistency is very high (alpha = ~.95), and it distinguishes ASD-affected individuals from controls with a Cohen’s d effect size of ~2.7 and from individuals with other psychiatric conditions with an effect size of ~1 [41]. The SRS-2 characterizes variation in the two DSM-5 domains of ASD: social communication and interaction and restricted interests and repetitive behaviors. Prior studies of the SRS-2 in clinical and epidemiological populations have established that these two subdomains encompass a unitary factor structure [29]. Guidelines for clinical interpretation of scores are described in detail in the SRS-2 manual; scores above 60T are in the range of clinical abnormality.

*Genomic Characterization (microarray) of WUSTL Sample*: In ASD probands and mothers from WUSTL clinics, buccal samples for chromosomal microarray analysis (CMA) were processed by the Lineagen commercial diagnostic laboratory using FirstStepDx PLUS, which incorporates 2,784,985 probes and is designed for use in patients with neurodevelopmental disorders. Chromosome Analysis Suite software (manufactured by Affymetrix) was used to interpret data [42]. Copy-number changes that were typically not reported included duplications of < 400 kb and deletions of < 50 kb, unless they were recurrent rearrangements with known associations with ASD or related disorders.

Buccal samples from research participants were processed through the Genome Technology Access Center (GTAC) at Washington University. GTAC used the CytoScan HD array (Thermo Fisher Scientific), which was designed with 2.6 million probes, including 1.9 million non-polymorphic probes selected for their linear response to copy number and genomic position. Copy number analysis was performed using the Chromosome Analysis Suite (version 3.2.0.1252 r10346) from Thermo Fisher Scientific. Copy-number changes that were typically not reported include duplications of <500 kb and deletions of <200 kb, unless these were known to be associated with clinical significance. Deletions < 500 kb that did not involve known genes were also not reported due to lack of supporting clinical evidence.

Analyses examined autosomal copy number variants and excluded X chromosome variants, given that (1) maternally inherited X-linked variants could incur greater male offspring vulnerability to ASD and would therefore represent less generalizable transgenerational risk across both sexes, (2) most implicated genetic risk variants for ASD are autosomal [43, 44], and (3) sex-linked inheritance profiles of ASD risk, with male sex as a predominant risk marker, do not pose the primary challenge to enhancing prediction of transgenerational recurrence.

### Data analysis

Analyses of each candidate predictor were conducted by comparing maternal profiles across families with contrasting patterns of intergenerational transmission of autism (e.g., simplex versus multiplex families). Descriptive statistics were calculated using SPSS. Student’s *t* tests, chi-square tests, and calculations of z-scores were used where appropriate to examine group differences, differences in group proportions, or differences in correlations. Effect sizes were estimated using Cohen’s d. Pearson’s correlation coefficients were used to evaluate bivariate associations. Coefficients of determination (*R*^2^) were used to estimate shared variance. In cases where prevalence rates in our samples were compared with general population prevalence, recently published statistics were used [17]. To standardize raw SRS-2 scores compiled from studies in which ratings were done using different forms (i.e., parental-, spousal-, or self-report), raw scores were converted to T-scores using the SRS-2 manual guidelines.

## Results

### Maternal Quantitative Autistic Traits (QATs) in simplex versus multiplex families (Analysis 1a)

We first explored the difference in phenotypic characteristics of women with a single child with ASD versus women with multiple ASD-affected children. We compared the QAT scores of mothers in simplex families from the Simons Simplex Collection (SSC; *n*=2839) and existing WU family studies (*n*=190) to multiplex families from the Autism Genetic Resource Exchange (AGRE; *n*=52) and existing WU family studies (*n*=55). Distributions of maternal SRS-2 T-scores were largely overlapping between the two groups, with mean scores for mothers from simplex families, 46.17 (SD = 7.29) only slightly lower than those from multiplex families, 47.14 (SD = 7.48). This difference was numerically in the expected direction but did not reach statistical significance (Table 2, Analysis 1a). We additionally tested whether the groups differed with respect to the proportion of mothers above and below an SRS-2 T-score cutoff of 45 for mild trait levels, and there was no significant difference between simplex and multiplex mothers (*x*^2^(1) = 0.36, *p* = .54).

**Table 2:** Maternal QAT burden stratified by family transmission pattern

ASD risk category	*N*	Mean (SD)	Statistics	Effect size (95% CI)
Analysis 1a. Maternal QATs in simplex versus multiplex families				
Simplex	3029	46.17 (7.29)	*t*(3134)=−1.36; *p*=.18	−0.13 (−0.33, 0.06)
Multiplex	107	47.14 (7.48)		
Analysis 1b. Maternal QATs in pedigrees consistent or not consistent with silent maternal transmission				
Pedigree not consistent with silent maternal transmission	73	46.63 (9.24)	*t*(94)=−1.06; *p*=.29	−0.25 (−0.72, 0.22)
Pedigree consistent with silent maternal transmission	23	48.96 (8.82)		
Analysis 2. Maternal QATs in simplex families with versus without de novo variant in ASD-affected offspring				
ASD-associated de novo variant	323	45.08 (6.52)	*t*(428)=−3.04, *p*=.003	−0.17 (−0.28, −0.05)
No ASD-associated variant	2528	46.27 (7.23)		
Analysis 3. Maternal QATs for unaffected sisters with an ASD-affected sibling and offspring with or without ASD				
No offspring ASD	33	41.33 (6.33)	*t*(31)=−2.20; *p*=.03	−0.85 (−1.63, −0.07)
Established offspring ASD	8	47.13 (8.06)		

*SD* standard deviation, *CI* confidence interval

### Maternal QATs in pedigrees consistent or not consistent with silent maternal transmission (Analysis 1b)

We next explored a more stringent manifestation of maternal carrier status for ASD risk to determine whether this was associated with differences in maternal QATs. In families from existing WUSTL studies (*n*=88) and the Second Generation Project (*n*=8), we compared SRS-2 T-scores between mothers of ASD-affected children with (*n*=23) versus without (*n*=73) a pedigree consistent with silent maternal transmission of ASD (Table 1). These pedigrees included mothers with a first-degree ASD-affected relative or two ASD-affected offspring who were maternal half-siblings. Mean SRS-2 scores were again numerically higher for the group suspected of silent maternal transmission but the difference did not reach statistical significance (Table 2, Analysis 1b).

### Maternal QATs in simplex families with versus without a de novo variant in ASD-affected offspring (Analysis 2)

**Fig. 1:**
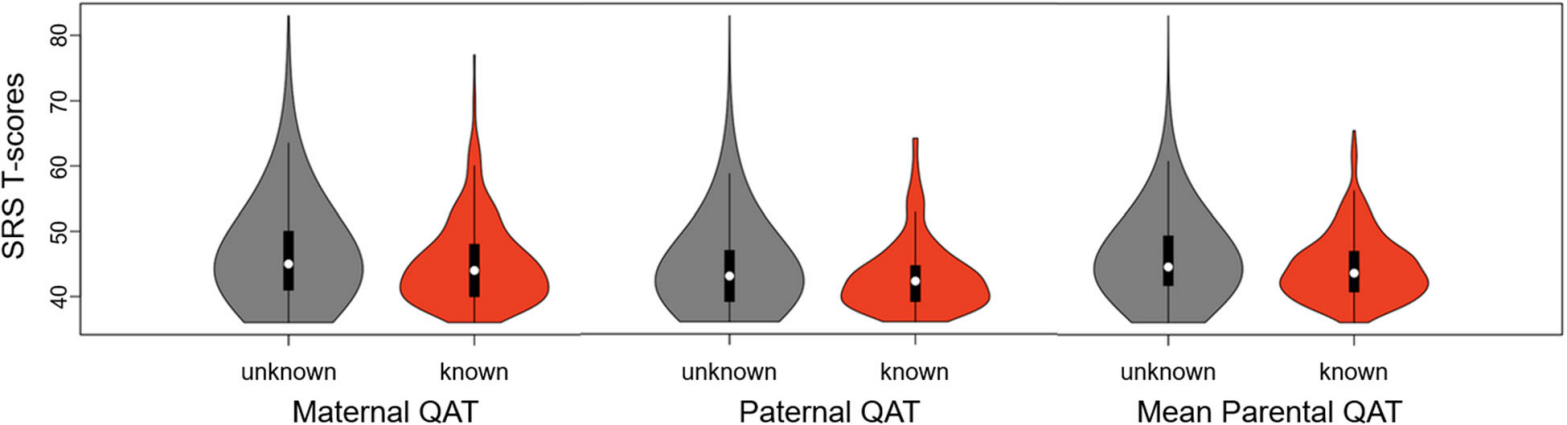
Mean parental QATs according to presence of ASD-associated pathogenic variants in offspring. QAT distributions for either mothers, fathers, or averaged parental SRS-2 scores have a slightly higher mean and wider range (encompassing higher scores) in families for which an ASD-affected child does not have known ASD-associated de novo variants, versus families in which the ASD-affected child has a known de novo variant. For parents of a child with known de novo variant, correlation of parental SRS-2 scores are as follows: *r*=0.26 (0.15, 0.36), *p*<.001; for parents of a child with unknown variants: *r*=0.31 (0.27, 0.34), *p*<.001)

We next examined whether maternal QAT burden was lower among mothers of ASD-affected children whose conditions were associated with de novo mutations. In participants from the SSC (Table 1), for whom one third of ASD cases would be expected to be at least partly influenced by a de novo pathogenic variant [45], we compared SRS-2 scores in mothers of an affected child with or without a known ASD-associated de novo variant (Fig. 1), as defined in the methods [36–38]. We hypothesized that in families with sporadic cases of ASD, lower familial ASD liability, as indexed by parental QATs on the SRS-2, would be observed for cases in which the proband carried a known ASD-associated de novo variant. As predicted, mean SRS-2 scores were significantly lower for mothers whose child had an ASD-associated *de novo* variant (*n*=323) versus mothers of children without such variants (*n*=2528; t(428)=−3.04, *p*=.003). Despite this highly significant result, the between-group difference had a small effect size (Table 2, Analysis 2). A parallel analysis based on SRS-2 scores in fathers (*n*=324 with known offspring variant, *n*=2527 without de novo variants) and mean parental SRS-2 scores for cases in which scores of both parents were available (*n*=321 with known offspring variant, *n*= 2517 without de novo variants) revealed a comparable result (Fig. 1).

### Maternal QATs for unaffected sisters with an ASD-affected sibling and offspring with or without ASD (Analysis 3)

In an independent sample from the Second Generation Project, we evaluated QATs in women who were sisters of individuals with ASD and mothers of one or more children (*n*=41 women from 40 families). We compared quantitative autistic trait burden of those with (*n*=8) versus those without (*n*=33) a child with diagnosed ASD. Among these sisters, those with ASD-affected offspring had higher SRS-2 scores, in this instance with a strong effect size for the difference (Table 2, Analysis 3).

### Offspring ASD diagnosis for unaffected mothers with an ASD-affected sibling (Analysis 4)

In addition to maternal QAT phenotyping, we obtained categorical data on offspring ASD diagnoses for 113 mothers with an ASD-affected sibling from 99 families in the Second Generation Project (Table 1). The total number of second-generation offspring in these families was 220, yielding an average of 1.9 children/family, which corresponds to the mean number of children per family observed in the U.S. in 2019 [46]. Comparable numbers of males and females among the second-generation offspring were consistent with an unbiased selection of families based on sex of the grandchildren. 13.2% of the children (19 of 111 boys and 10 of 109 girls) had a community ASD diagnosis, a greater percentage than the general population prevalence [17] of 1.9% (*x*^2^(1) = 18.87, *p* <.001).

### Maternally inherited variants in ASD-affected offspring with or without maternal family history of ASD (analysis 5)

To investigate associations between familial history of ASD and inherited genetic risk, we compared rates of maternally inherited chromosomal rearrangements in families with sporadic versus familial forms of ASD. Specifically, proportions of maternally inherited variants were compared across families in which mothers of ASD-affected children were (1) unrelated to an individual with autism (*n*=73) and (2) had a prior family history of ASD (*n*=30) (Table 1). In the group without a maternal family history of ASD, four of 73 children with ASD (6%) had maternally inherited chromosomal rearrangements, all variants of unknown significance. In the group with a maternal family history of ASD, five (16%) were found to have maternally inherited rearrangements, all variants of unknown significance, with the percentage difference not being significant (*x*^2^(1) = 2.08, *p* = .15).

### Parental age in simplex families relative to the number of de novo variants in ASD-affected offspring and their unaffected siblings (analysis 6)

As noted above, older parental age is associated with increased likelihood of de novo variants associated with neurodevelopmental disorders [23], and paternal age has been observed to correlate with the number of de novo variants in ASD-affected offspring [2, 23, 47]. We tested for confirmation of the relationship between parental age and number of ASD-associated de novo variants that were reported in the SSC, using the classification of de novo variants applied in analyses above (Table 1). Because the SSC is limited to families with sporadic cases of ASD, it would be expected to be enriched for such variants. We also compared the correlation between parental age and de novo variants for ASD probands and the probands’ unaffected siblings in the SSC to evaluate whether the hypothesized association was specific to offspring affected with ASD in this sample.

**Fig. 2:**
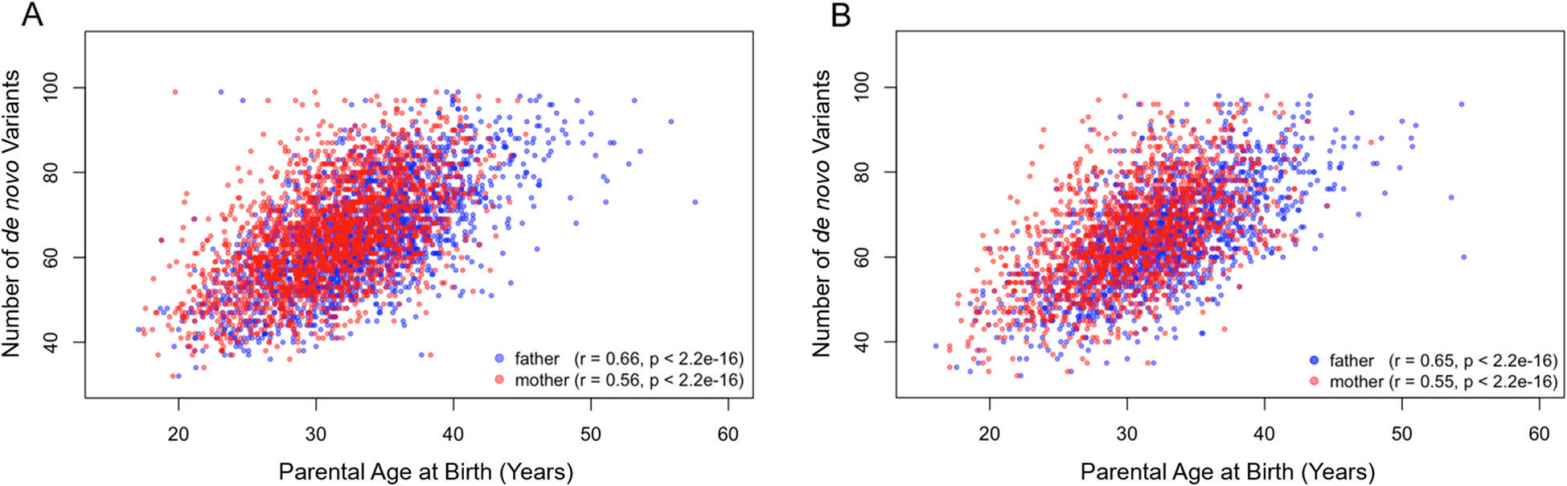
Relationship of maternal and paternal age to offspring de novo variant burden. Parental age at the time of birth of a child with ASD (**a**) or an unaffected sibling (**b**) is plotted in relationship to the number of ASD-associated de novo variants in these offspring. Similar correlations are observed for both ASD-affected and unaffected offspring, suggesting the observed relationship is not specific to ASD

Among both mothers and fathers (*n*=2140), increasing age was associated with a higher number of de novo variants in ASD-affected children (Fig. 2). A strong correlation was observed for paternal age, *r*=0.66 (95% CI=0.64, 0.68), *p* <.001), accounting for 44% of variance in the number of de novo variants in ASD-affected offspring. The correlation was slightly lower for mothers, *r*=0.56 (95% CI=0.53, 0.59), *p* <.001, accounting for 31% of the variance in de novo variant number, and this difference from fathers was significant (*z*=5.23, p <.001). Similar paternal and maternal age correlations, each with overlapping confidence intervals, were also observed for number of de novo variants in unaffected siblings of ASD probands (*n*=1605); for paternal age, *r*=0.65 (95% CI=0.62, 0.68), *p* <.001, and for maternal age, *r*=0.55 (95% CI=0.51, 0.58), *p* <.001, indicating that parental age accounted for a similar level of variance in de novo variants in offspring from simplex families irrespective of an ASD diagnosis.

## Discussion

Empowering families affected by autism to understand and recognize parameters of increased risk to second-generation offspring has important translational implications, both for reproductive health planning, including pre-conceptional genetic counseling, and for enhancing vigilance for the earliest detection and developmental intervention for ASD-affected offspring. In this study, we leveraged a diverse array of new and existing research datasets to examine four candidate predictors ascertainable in the parental generation: (1) elevation in sub clinical autistic traits, which are known to aggregate in the close family members of individuals with ASD; (2) parental history of a sibling with ASD, (3) autosomal inherited copy number variants detectable by clinical genotyping, and (4) parental age in relation to risk for germline variation that would influence risk among offspring. In the USA, ASD affects one in 54 children [17], a prevalence estimate that has risen from one in 110 children over the past decade [48]. Consequently, the scenario of prospective parenthood for the unaffected siblings of individuals with autism is experienced by a very large segment of the nation’s population, including hundreds of thousands of women of child-bearing age at the present time. We know of no prior published studies that address the clinical specification of transmission risk for ASD in such common scenarios, except for highly focused studies on the transmission of rare inherited genetic conditions (e.g., 16p11.2 deletion, Fragile X Syndrome) [49–51], which collectively account for approximately five percent of all cases of autism in the population [9].

Recently, using data from a large epidemiologic multigenerational cohort, we provided the first robust estimates of the *average* risk of autism to offspring of men and women with siblings affected by autism, indicating a 2–3 fold elevation in risk over the mean for the population [18, 52]. In that study, there were no available data on characteristics of the parents that might have allowed recognition of higher or lower individual-level transgenerational risk, relative to the mean, within individual families. In our analyses of candidate parental predictors, we observed that although measurable variation in these characteristics corresponded to hypothesized relationships with ASD’s genetic risk architecture, none of these candidates alone could be considered adequate to refine estimates of the average risk to offspring of unaffected siblings of individuals with ASD. For example, the presence of a pathogenic de novo variant in an ASD proband within a simplex family was associated with lower parental QATs compared to families in which the proband lacked such a variant (Fig. 1). This statistically significant difference aligned with our hypothesis that ASD cases attributable to a de novo variant would be associated with a lower burden of heritable ASD risk variants in the parents, although the small effect sizes observed here, and across other QAT phenotypic contrasts (Table 2), were not commensurate with the ability to clinically resolve individual parental risk. Nonetheless, there are important implications for these results, which provide a critical starting point for future efforts toward clinical parameterization of ASD risk to second-generation offspring within affected families.

A first implication, based on findings in the Second Generation Study dataset, is that offspring prevalence of ASD among sisters of individuals with ASD is measurably greater than the general population. These results were in keeping with our recent epidemiologic study of over 800,000 individuals in a two-generation cohort from the Swedish National Register, in which we reported a 3–5% recurrence risk of ASD in siblings of an individual with ASD [18]. Although the percentage observed in our quasi-prospective sample, 13%, was numerically higher, this estimate should be considered cautiously given the much smaller sample and comparatively strong ascertainment biases. Ongoing tracking of the latest generation in ASD-affected families is nevertheless warranted as an uptick in average risk may reflect the possibility that societal trends, e.g., increasingly accessible social networking opportunities through the internet or social media, could enhance preferential mating in relation to QATs [19, 20, 53], which has been linked to amplification of QATs across generations [19].

At the level of maternal QATs in the Second Generation Project, we further observed that sisters of individuals with ASD who manifest sub clinical autistic traits are at increased risk for transmission of ASD liability to their offspring when compared to sisters without such trait elevations. This result, together with our findings on offspring diagnoses above, is congruent with offspring ASD risk estimates based on a prior intergenerational quantitative trait study. In that study, which incorporated all families in an epidemiologic sample, not just those affected by autism [19], *relative* risk of ASD was increased by 52.0% (i.e., from 1 to 1.52%) among children for whom either parent demonstrated elevations in quantitative autistic trait scores at the upper quintile of the population distribution and by 85.0% when parents’ scores were concordantly elevated. The similar increment in risk conferred by elevated QATs in each additional parent is consistent with additive rather than multiplicative transmission of risk, an observation reinforced by ASD recurrence rates from our studies of second-generation ASD outcomes (see summary, Table 3). Thus, findings in this study and the literature collectively support enhanced developmental surveillance in at-risk families while also mitigating concerns for amplification of offspring ASD risk that might be predicted under the assumptions of the female protective effect hypothesis [30].

**Table 3:** Summary of recurrence risk estimates for prospective parents relative to general population

Indicator of familial ASD liability	Relative recurrence risk	Source
Mother with ASD-affected sibling*	3	Bai D, et al. [18]
Father with ASD-affected sibling*	2	
Mother and father with upper quintile of QATs	1.85	Lyall K, et al. [19])
Either mother or father with upper quintile of QATs	1.52	
Mother with ASD-affected sibling* and elevated QATs	[~6.5]**	Second Generation Project

*Idiopathic ASD is assumed. For ASD with a known genetic cause, recurrence will vary based on that variant’s inheritance and penetrance. For example, in Renpenning Syndrome, an X-linked disorder affecting males [54], a sister carrying the associated X-linked mutation has a 50% likelihood of having an affected son, who then has an estimated 38% likelihood of ASD [55].

**This estimate, based on dividing our observed second-generation offspring ASD prevalence (13%) by general population ASD prevalence (~2%), is highly preliminary, given it is derived from a small sample subject to bias from clinical ascertainment of ASD. Nevertheless, it confirms elevated transgenerational ASD risk in parents with two markers of aggregated ASD liability (having an ASD-affected sibling and elevated QATs) and highlights the need for future research in large, genetically informative samples examining joint interactions of predictors of transgenerational ASD risk

Second, we did not observe a disproportionate aggregation of abnormal maternally inherited chromosomal rearrangements among mothers of ASD-affected children based on the presence or absence of a maternal family history of ASD. The proportions in both groups were substantial (16 and 6 percent, respectively) but were comprised mostly of variants of uncertain significance in relation to the pathogenesis of autism. Genomic characterization of unaffected siblings in these families, as well as clinical and developmental phenotyping, remains an important future direction to evaluate whether these variants are consistently associated with ASD or other alterations in neurodevelopment, given genetic overlap among neurodevelopmental disorders [11, 12] and elevated rates of psychopathology-related concerns in unaffected siblings of children with ASD [56]. Our observation of maternally inherited variants of unknown significance also suggests that among unaffected adults of child-bearing age, the presence of an ASD-affected family member would not yet constitute a trigger for routine screening for a chromosomal rearrangement *unless a heritable pathogenic variant has been identified in the affected family member*. As recommended by the American College of Medical Genetics [57], all individuals affected by autism should undergo clinical genomic screening. When an affected individual in a family tests positive for an inherited mutation, however, this constitutes opportunity for actionable pre-conceptional testing of first-degree relatives of child-bearing age (depending upon the penetrance of the mutation) given the high likelihood of transmission from a carrier to his/her offspring. As knowledge about pathogenicity of genomic variation improves, the relatively high rates that we observed here may signal future opportunity for genomic characterization of prospective parents to individually refine estimations of transgenerational risk. This screening is likely to include polygenic risk scores, as well as ascertainment for chromosomal rearrangements, autosomal single nucleotide variants, or rare recessive sequence variants of major effect, particularly in the situation when an inherited condition mediated by such variants, including those on a sex chromosome, have been identified in an ASD-affected family member.

The third implication of these results is that advancing parental age has a demonstrable effect on the likelihood that an offspring of a parent will harbor a de novo variant that has been associated with a neurodevelopmental disorder, a result corresponding to prior studies [2, 12, 46]. The relationship between parental age and de novo variants, notable for both parents, was stronger for fathers than mothers, consistent with hypotheses regarding the increased frequency of germ cell mutations as men age [27, 28] and prior population-based studies [25, 26]. Importantly, a similar increase in these de novo variants with parental age was observed both for ASD-affected offspring and their unaffected siblings. Thus, while advancing parental age appears to contribute to genetic factors linked to increase risk of ASD, this effect is not sufficiently specific to differentiate offspring risk of ASD and is likely influenced by sibling variations in family genetic background or in the de novo variants themselves, which are not necessarily shared by siblings [22]. An immediate priority of future research should therefore be to translate the incremental effects of parental age on offspring risk when additional risk factors are present.

Since de novo variants cannot generally be detected or predicted by assays in the tissues of parents (with the exception of in utero recovery of offspring DNA from either amniocentesis or the analysis of cell-free DNA in blood samples of pregnant women), the offspring risks incurred by advanced paternal and maternal age are important to be aware of, given steady increases in average parental age over several decades [58]. Practice guidelines for medical geneticists and obstetricians have noted the association of paternal age with ASD and other neurodevelopmental and genetic disorders in offspring [59, 60], yet few websites devoted to the prevention of birth defects, including those of the March of Dimes and The Centers for Disease Control, describe any risk other than that previously referred to as “advanced maternal age,” resulting in a missed opportunity to inform the public of existing evidence indicating offspring risk for older fathers. Going forward, we advocate for use of the more representative term “advanced parental age” to promote awareness that older age in fathers as well as mothers is associated with higher offspring rates of genetic and neurodevelopmental conditions, so that prospective parents can make educated reproductive choices. Targeting educational campaigns to demographics at highest risk of older parenting seems prudent, and this information should be widely disseminated to individuals across all socioeconomic groups contemplating additional pregnancies.

There are significant limitations of these data, which were largely derived from research datasets that were designed for other purposes. For a number of the analyses, the statistical power was constrained on the basis of limitations in sample size, but was not so limited that we would have been unable to detect shifts in risk that would be actionable in a genetic counseling sense (e.g., a rise in the level of ASD risk to offspring one order of magnitude greater than general population risk). None of our data were obtained within an epidemiologic sampling frame and were therefore vulnerable to the usual effects of clinical ascertainment bias. In our analyses testing associations between parental age and offspring de novo mutations, we were unable to incorporate phasing to account for the parent-of-origin relative to de novo variants, an approach planned for future related studies. While the magnitude of our observed correlations for maternal and paternal age could thus be somewhat inflated, our finding of higher correlations between paternal versus maternal age and de novo mutations is consistent with work including phasing [61]. The data collections also did not afford the opportunity to comprehensively examine joint risk of the candidate predictors that we explored, rather data were typically available for only one predictor in a given parent. Despite these considerable limitations, the importance of clarifying the predictive power of these readily-obtainable measurements of parents among the hundreds of thousands of prospective parents whose families are affected by autism in the USA warranted thorough examination of the available data, which has served to parameterize the effect of individual predictors in families with contrasting transmission patterns for idiopathic ASD.

## Conclusions

To our knowledge, this is the first empirical test of several hypothetical predictors of individual-specific parental risk for the intergenerational transmission of autism. We conclude that, when considered individually, none reach thresholds for actionability for pre-conceptional counseling. Specifically, individual estimation of risk to offspring of adults in ASD-affected families is not straightforwardly improved by ascertainment of parental phenotype or family history, and it is not yet clear whether genomic screening of prospective parents in families affected by idiopathic ASD is warranted as a clinical standard. These data support prior observation [18] of significant elevation of ASD risk among the offspring of unaffected siblings of individuals with autism. Systematic screening of affected family members for heritable pathogenic variants, including rare sex-linked mutations, will identify a subset of families in whom risk to second-generation offspring is individually specifiable and substantially elevated. Polygenic risk scores are only weakly predictive at this time [62] but steadily improving and ultimately may enable more robust prediction either singly, or when combined with the risk variables examined in this study. With future research and development of more predictive, comprehensive risk assessments, pre-conceptional genetic counseling will provide the opportunity for personalized risk estimations as well as psychoeducation to promote timely surveillance strategies for the second generation.

## Data Availability

The datasets used for the current study are available from senior author J.N.C. on reasonable request.

## Abbreviations

ASD: Autism spectrum disorder
PGR: Polygenic risk
QATs: Quantitative autistic traits
IAN: Interactive Autism Network
TKI: Telethon Kids Institute
TEACCH: Treatment and Education of Autistic and Related Communication Handicapped Children
WUSTL: Washington University in St. Louis
SRS-2: Social Responsiveness Scale-2
AGRE: Autism Genetic Resource Exchange
SSC: Simons Simplex Collection
SPSS: Statistical Package for the Social Sciences
